# Implementation gaps of the Portuguese National Health Plan: a stakeholders’ perception analysis: Alexandra Costa

**DOI:** 10.1093/eurpub/ckac130.165

**Published:** 2022-10-25

**Authors:** A Costa, A Rosa, L Costa, R Mexia, CM Dias, T Caldas de Almeida

**Affiliations:** 1 Department of Health Promotion and NCD Prevention, National Institute of Health Doutor Ricardo Jorge, Lisbon, Portugal; 2 Institute of Social and Political Sciences, University of Lisbon, Lisbon, Portugal; 3 Department of Epidemiology, National Institute of Health Doutor Ricardo Jorge, Lisbon, Portugal; 4 BioISI-Biosystems & Integrative Sciences Institute, Faculty of Sciences, University of Lisbon, Lisbon, Portugal

## Abstract

**Background:**

The Portuguese National Health Plan (PNS) 2012-2016, extended to 2020, was the strategic health document shaping the direction of intervention within the Health System. The National Institute of Health Doutor Ricardo Jorge (INSA) is the institution responsible for carrying out its final evaluation. To underpin PNS final evaluation a multistep process was designed.

**Methods:**

A mixed methods study using a convenience sample was implemented to assess stakeholders’ perception about five PNS dimensions: dissemination, communication, implementation, impact, and evaluation. For this purpose, two different tools were used. Semi-structured interviews with former health policy managers were conducted. In addition, an online survey was designed and widely distributed to additional stakeholders. From October 2019 to February 2020, data was collected using two separate instruments. Integral transcriptions of the interviews were made. Qualitative content analysis and quantitative descriptive analysis were used.

**Results:**

A total of 12 interviews and 179 valid surveys were obtained. Regarding stakeholders’ perception about PNS implementation process, there was an overall positive recognition about the strategic and operational relevance of the PNS, as a common dialogue platform and a tool for health improvements both for health status and health system function in Portugal. Among pointed implementation barriers, management was mentioned as the major constraint, mainly due to shortage of human and financial resources to carry out the recommended interventions within the PNS timeline.

**Conclusions:**

As part of the evaluation process the research team found relevant gain knowledge of the wider context in which PNS was developed and implemented, based on stakeholders’ perception. Their considerations are important not only to support the definition of the questions and criteria for PNS final evaluation, but also to highlight key issues for the future policy cycle.

**Key messages:**

- Gain knowledge about the wider context in which the Portuguese National Health Plan (PNS) was developed and implemented was considered a crucial step towards its final evaluation.
- Stakeholders’ perception about PNS can inform about implementation gaps, key enablers and barriers and further support the definition of the questions and criteria for PNS final evaluation.

